# 360-Degree Sclerosed Vessels and Candle Wax-Like Deposits as an Atypical Exudative Presentation of Aggressive Retinopathy of Prematurity in a Neonate With Severe Anaemia

**DOI:** 10.7759/cureus.103623

**Published:** 2026-02-14

**Authors:** Sindhuja Srinivasan, Akash Belenje, Subhadra Jalali

**Affiliations:** 1 Srimati Kanuri Santhamma Center for Vitreo Retinal Diseases, Anant Bajaj Retina Institute, Standard Chartered-LVPEI Academy for Eye Care Education, Kallam Anji Reddy Campus, L V Prasad Eye Institute, Hyderabad, IND

**Keywords:** aggressive retinopathy of prematurity, atypical presentations of rop, candle wax-like preretinal deposits, exudative a-rop, neonatal anaemia, sclerosed vessels

## Abstract

We report a finding of 360-degree completely sclerosed vessels presenting in aggressive retinopathy of prematurity (A-ROP) in a neonate with severe anaemia. A one-month-old baby with a history of neonatal intensive care unit (NICU) admission for respiratory distress and multiple blood transfusions for systemic anaemia was referred to our hospital with A-ROP. Posterior pole examination revealed candle wax-like exudative deposits along the retinal vessels, with areas of early vascular sclerosis. Ultrawide field fundus photo documentation for both eyes at various presentations, including after the anti-VEGF injections in both eyes, along with laboratory investigations for the systemic evaluation of the baby, was performed. The baby received intravitreal anti-VEGF therapy and was subsequently lost to follow-up. The baby returned three months later and presented with severe systemic anaemia and complete 360-degree vascular sclerosis involving all quadrants, an exceedingly rare and striking presentation. Following prompt correction of the anaemia with blood transfusions, there was dramatic reperfusion of the retinal vasculature, though significant peripheral avascular retina persisted. Laser photocoagulation was performed to treat the persistent avascular retina (PAR). Neonates presenting with ROP and vasculitis-like vascular sheathing may harbour underlying, uncorrected anaemia, warranting thorough systemic evaluation. This highlights the importance of comprehensive monitoring, including detailed retinal vascular assessment and evaluation of haemoglobin levels, thereby preventing progression to extensive vascular sclerosis, as seen in this patient.

## Introduction

The global burden of retinopathy of prematurity (ROP) has increased substantially over the past two decades, emerging as a major cause of preventable childhood blindness worldwide [1]. With many recent advances in neonatal care, the survival of extremely preterm and low-birth-weight infants has improved. However, these infants remain at the greatest risk for developing severe ROP. ROP classically progresses from stages 1 to 5, except for aggressive retinopathy of prematurity (A-ROP), which can progress rapidly, skipping stages [1]. Sanghi et al. described Hybrid ROP in their study, where they observed a few cases that had features suggestive of both A-ROP and staged ROP co-existing in the same eye [1,2]. Three patterns of hybrid ROP have been described in the literature: I Ridge at the junction of the vascular and avascular retina; II Ridge in the vascularised retina; III Poorly defined ridge close to the optic disc, with the vitreous harbouring mat-like fibrous proliferation [1,2]. The exudative retinal detachment (ERD) in ROP that at times occurs after extensive laser photocoagulation is mainly attributed to inflammation [2,3]. In cases presenting with either chronic traction or an acute bleb-like configuration of tractional detachment, we see a combination of both tractional and exudative retinal detachment components [4,5]. Isolated ERD without a tractional component nonetheless differs from the typical presentation of staged ROP and/or A-ROP, as it is also a rare initial presentation of ROP [4,5]. The clinical features and outcomes of such presentations are sparse in the literature, and due to their rarity, the pathogenic mechanisms associated with them are largely unknown.

Anaemia is a well-recognised systemic risk factor for the development and progression of ROP in neonates. It commonly arises from several factors, such as an immature haematopoietic system, inadequate erythropoietin production, failure to thrive, iatrogenic blood loss due to frequent sampling, postnatal infections, and suboptimal maternal health factors such as maternal anaemia and malnutrition [6-8]. Neonatal anaemia is defined as a haemoglobin level at least two standard deviations below the mean for a given gestational or chronological age and may result from multiple aetiologies that reduce red blood cell mass [6-8]. As a modifiable risk factor, anaemia has been consistently associated with an increased risk of ROP development [6-8]. Evidence suggests that timely correction of anaemia, particularly through blood transfusion, plays a critical role in initiating regression of ROP [7,8]. According to World Health Organisation criteria, anaemia severity in infants is categorised as mild (haemoglobin 10.0-10.9 g/dL), moderate (7.0-9.9 g/dL), or severe (<7.0 g/dL) [6-8].

We describe an atypical presentation of ROP in posterior Zone I, exhibiting features consistent with hybrid ROP and characterised by unusually extensive candle wax-like preretinal deposits located both within the vascularised retina and along the vascular-avascular junction. These deposits demonstrated dramatic resolution within one to two weeks following intravitreal administration of anti-vascular endothelial growth factor (anti-VEGF) therapy. However, post-injection, with the systemic anaemia left uncorrected, the disease unfortunately progressed to total sclerosis of all the vessels. This was promptly managed with blood transfusion and laser indirect ophthalmoscopy (LIO) to the final persistent avascular retina (PAR).

## Case presentation

Case description

A one-month-old neonate born to parents with second-degree consanguinity was delivered preterm at 28 weeks of gestation via caesarean section, with a birth weight of 1100 grams. The mother was a 22-year-old primigravida. She was moderately built and nourished. Following delivery, the neonate required admission to the Neonatal Intensive Care Unit (NICU) for 30 days due to respiratory distress syndrome and recurrent apnoeic episodes. During the NICU stay, the neonate received oxygen supplementation administered via oxygen hood for three weeks, followed by nasal prongs for one week. The neonate received surfactant for the treatment of respiratory distress. The neonate was managed for low birth weight and underwent two blood transfusions during the admission period for severe anaemia (haemoglobin 6.0 g/dL) and thrombocytopenia (125 × 10³/mm³). The perinatal history is significant for extreme prematurity, prolonged oxygen exposure, and multiple transfusions, thereby collectively placing the infant at high risk for retinopathy of prematurity.

**Figure 1:**
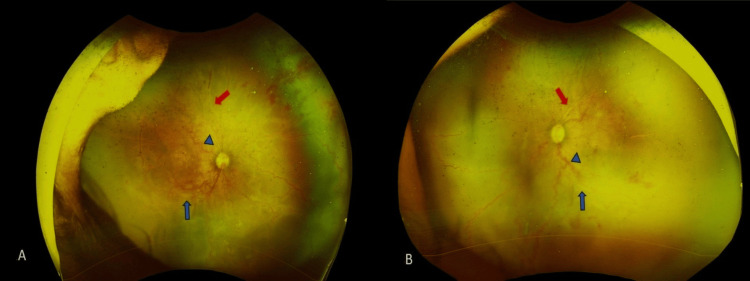
Ultra-widefield fundus image of both eyes Optos ultra-widefield images of the right eye (A) and left eye (B) fundus show retinal vascular tortuosity (arrowhead), extensive candle wax-like preretinal deposits with sheathing of vessels extending from the vascular-avascular junction in both eyes (blue arrows), and sclerosed vessels in both eyes (red arrows).

At presentation to our eye institute, the infant weighed 1300 grams, and laboratory evaluation at our institute revealed a haemoglobin level of 7.0 g/dL. Ophthalmic examination of both eyes revealed clear media and the presence of arterio-venous loops with deposits around the vessels and the ridge. Ultra-widefield fundus imaging on Optos showed significant tortuosity of the posterior Zone I retinal vessels. The arteries and veins appeared similar in calibre and configuration, with multiple arteriovenous shunts noted at different levels. Extensive candle wax-like preretinal deposits were noted, stretching from the posterior pole to the vascular-avascular junction (Figure 1: A, B). These deposits appeared very confluent and prominent. Based on these clinical and imaging findings, a diagnosis of A-ROP, Zone I, in both eyes was established, accompanied by vasculitis-like vascular changes considered likely secondary to significant systemic anaemia.

Treatment

The infant was advised an injection of intravitreal anti-VEGF Bevacizumab, half of the adult dose (0.625 mg/0.025 mL), which was delivered on the same day in both eyes after obtaining necessary parental consent for the management of A-ROP. The possible ocular and systemic side effects associated with the procedure, the requirement for bedside injection as the baby was admitted in a paediatric setup, and the need for prolonged and frequent follow-up were explained to the parents. The possibility of disease reactivation and the need for any additional treatment in such a scenario were also explained. Arrangements for blood transfusion were coordinated at an appropriate paediatric facility. A follow-up visit was scheduled two weeks later.

Outcome and follow-up

**Figure 2:**
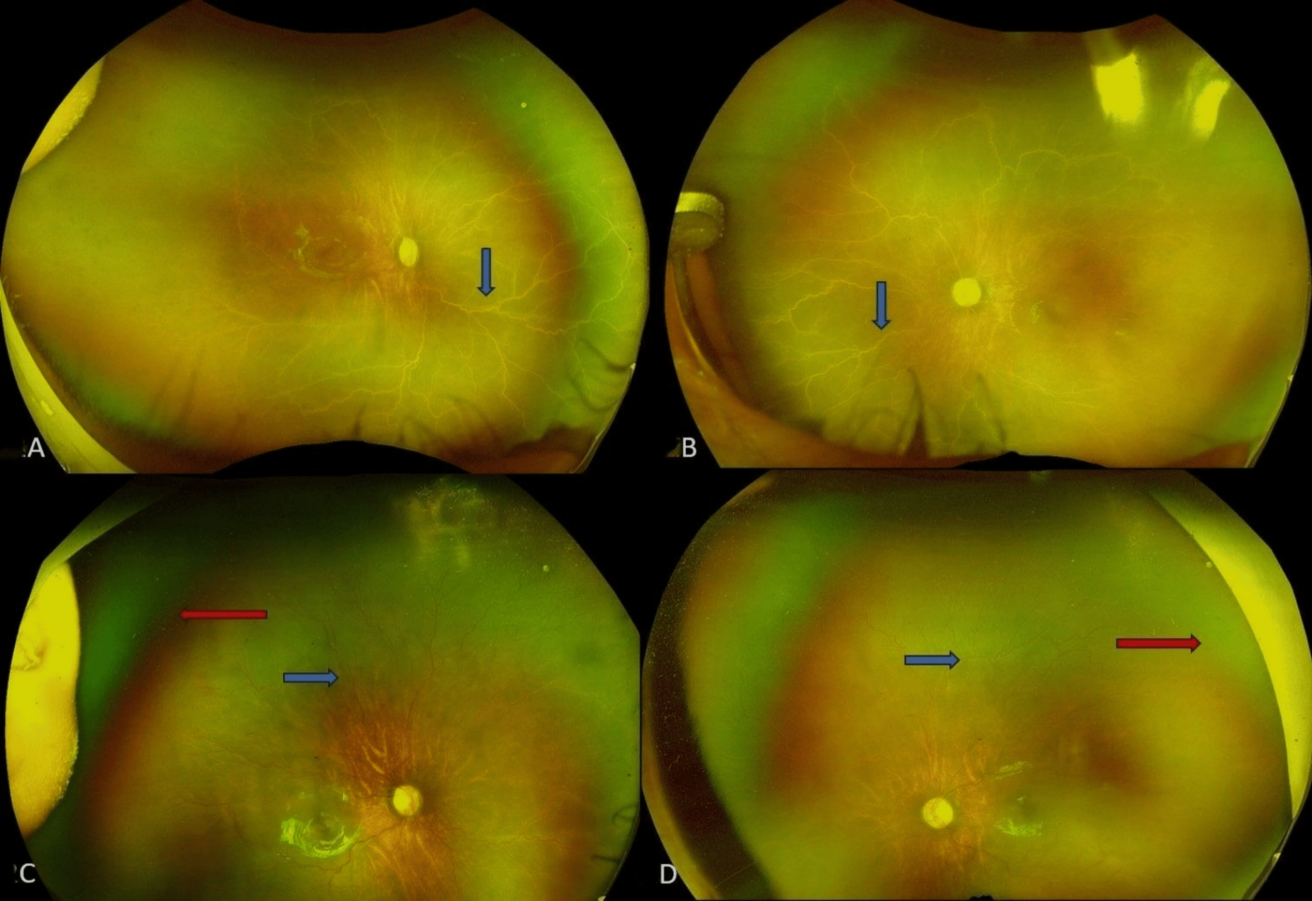
Ultra-widefield fundus image of both eyes Optos ultra-widefield images of the right eye (A) and left eye (B) fundus show 360-degree complete vascular sclerosis of all the vessels in both eyes (blue arrow) after three months of loss to follow-up following the initial anti-VEGF injection. The right eye (C) and left eye (D) show reperfusion of vessels (blue arrow) one month after blood transfusion, with peripheral persistent avascular areas in both eyes (red arrow).

At the two-week follow-up, the vasculature in both eyes started to improve, the preretinal deposits dramatically resolved, and a reduction in the plus component was observed. In the subsequent follow-ups, there was no trace of these preretinal deposits around the vessels. The baby was then lost to follow-up for three months and was reviewed later after being referred to us again by a local paediatrician. Examination of the fundus during that visit revealed complete white sclerosis of all the vessels in both eyes (Figures 2: A, B), and the baby had a haemoglobin level of 6.5 g/dL. We referred the baby to the nearest paediatrician immediately for blood transfusions and management of the anaemia and reviewed him with an update after two weeks, when the haemoglobin level improved to 9 g/dL and the vessels were perfused normally again up to anterior Zone 2 (Figure 2: C, D), beyond which the retina remained avascular.

Considering the systemic improvement and arrest of vascularisation beyond anterior Zone 2, we decided to perform laser to the PAR and followed the baby. The last follow-up showed well-regressed ROP with laser marks. This would not have been possible without suspicion of vascular sclerosis secondary to anaemia and prompt blood transfusion, thereby treating the underlying cause.

## Discussion

The case of A-ROP presented here exhibited an atypical presentation that clinicians must recognise to prevent delays in diagnosis and treatment. To the best of our knowledge, based on an extensive literature search, this appears to be the first reported case of its kind. Unfortunately, lack of attention to the infant’s haemoglobin status and failure to ensure timely follow-up resulted in a drop in haemoglobin to 6.5 g/dL and subsequent complete retinal vascular sclerosis, a potentially devastating outcome that could otherwise have been avoided. We describe an unusual presentation of posterior Zone 1 A-ROP, accompanied by extensive candle wax-like preretinal deposits. These deposits were observed not just at the vascular-avascular junction but also within the vascularised posterior retina, an uncommon distribution. The deposits responded and resolved dramatically within two weeks of intravitreal anti-VEGF (Bevacizumab) therapy. However, recurrence of the disease was noted with complete vascular sclerosis in association with uncorrected systemic anaemia. Following blood transfusion, the retinal vasculature regained normal perfusion within one week, although further physiological vascularisation failed to progress. Given the PAR, laser photocoagulation was subsequently planned as the definitive management.

Popcorn lesions refer to new vessels noted intraretinally, which, when fused, grow posteriorly from the ridge [9-11]. In our case, the exudates were too extensive, which is unlikely to be seen along with popcorn lesions [1,12]. Sometimes, these popcorn-like deposits or "popcorn lesions" may resolve following laser treatment, although not as rapidly as observed in our case. On the other hand, endogenous endophthalmitis can occasionally present with exudates, but it was ruled out in our scenario as the eye was quiet with no activity, the vitreous was clear, and there was a good therapeutic response to anti-VEGF therapy. Chronic organised preretinal haemorrhage may mimic a similar appearance, but it typically contains at least some fresh red blood components, which were absent here. Fibrovascular proliferation, particularly when the fibrous component predominates over the vascular component, can also resemble these lesions; however, such membranes are usually located along the posterior border of the ridge and exhibit some degree of vascularity, features not seen in our case, where the deposits were entirely avascular on clinical examination. Another differential consideration is extensive vitreous condensation over the vascularised retina; however, in our patient, the deposits were situated directly on the retinal surface rather than within the vitreous cavity. Pattern III hybrid ROP that had mat-like opaque fibrous membranes, as described by Sanghi et al. [1] or in the canine model of oxygen-induced retinopathy, had similarity to the morphology described here [12]. Hartnett et al. believed that oxygen toxicity could result in poorly formed retinal vessels and a blood-retinal barrier, which could eventually lead to abnormal fibrin or exudation within the vascularised retina. ROP occurs with improper vasculogenesis and/or angiogenesis. Severely disorganised retinal vessels in A-ROP, in association with intertwining areas of avascularity, can lead to abnormal exudation observed both subretinally and perivascularly [13]. In the presence of extensive choroidal ischaemia, fibrinoid necrosis of choroidal arterioles results in disruption of the outer retinal barrier, leading to exudative detachment [14].

The finest quality of the retinal vessels is their translucency, which is usually the first to disappear when there is any pathological insult to them [15]. The drastic response of the exudates following anti-VEGF therapy suggests the possibility of a strong VEGF-driven pathogenic mechanism. Additionally, compromised systemic factors such as anaemia, sepsis, apnoea, and inadequate weight gain may have further contributed to the development and progression of these retinal changes. Although limited reports exist describing partial vascular sclerosis in atypical ROP cases associated with exudative retinal detachment, this case is distinctive in demonstrating complete 360-degree vascular sclerosis of all posterior pole vessels, followed by remarkable restoration of perfusion after correction of anaemia through blood transfusion. This unique sequence of events shows the importance of considering systemic contributors, particularly severe anaemia, in cases of atypical or rapidly evolving ROP. Documentation and analysis of similar cases in the future will be crucial for improving our understanding of the relationship between the degree of anaemia, the extent of vascular sclerosis, and the natural course of recovery with timely systemic and ocular intervention.

## Conclusions

This case illustrates a rare presentation of 360-degree complete retinal vascular sclerosis in a premature infant with significant anaemia, which demonstrated substantial reversal following blood transfusion. This observation emphasises the essential role of timely identification and correction of anaemia in the management of A-ROP. Furthermore, this case raises the possibility that infants presenting with ROP and vasculitis-like vascular sclerosis may harbour underlying, uncorrected anaemia, warranting thorough systemic evaluation. It highlights the importance of comprehensive monitoring that includes not only detailed retinal vascular assessment but also regular evaluation of haemoglobin levels, thereby preventing progression to extensive vascular sclerosis, as seen in this patient. In this context, prompt referral to a paediatrician, early detection of sclerosed vessels, and immediate initiation of blood transfusion were key determinants of good outcomes.

## References

[1] Sanghi G, Dogra MR, Katoch D, Gupta A (2014). Aggressive posterior retinopathy of prematurity in infants ≥ 1500 g birth weight. Indian J Ophthalmol.

[2] Ehmann D, Greve M (2014). Intravitreal bevacizumab for exudative retinal detachment post laser therapy for retinopathy of prematurity. Can J Ophthalmol.

[3] Moshfeghi DM, Silva RA, Berrocal AM (2014). Exudative retinal detachment following photocoagulation in older premature infants for retinopathy of prematurity: description and management. Retina.

[4] Hovland P, Mukai S, Hirose T (2008). Advanced retinopathy of prematurity. Albert & Jakobiec’s Principles & Practice of Ophthalmology.

[5] Patel A, Padhy SK, Saoji K (2021). Bleb-like posterior combined retinal detachment in severe retinopathy of prematurity: clinical characteristics, management challenges and outcome. Eye (Lond).

[6] Bain A, Blackburn S (2004). Issues in transfusing preterm infants in the NICU. J Perinat Neonatal Nurs.

[7] Gaynon MW (2006). Rethinking STOP-ROP: is it worthwhile trying to modulate excessive VEGF levels in prethreshold ROP eyes by systemic intervention? A review of the role of oxygen, light adaptation state, and anemia in prethreshold ROP. Retina.

[8] Banerjee J, Asamoah FK, Singhvi D, Kwan AW, Morris JK, Aladangady N (2015). Haemoglobin level at birth is associated with short term outcomes and mortality in preterm infants. BMC Med.

[9] Singh R, Chaudhary N, Jassar R (2022). Neonatal Anemia. Newborn.

[10] Wallace DK, Kylstra JA, Greenman DB (1998). Significance of isolated neovascular tufts (“Popcorn”) in retinopathy of prematurity. J Am Assoc Pediatr Ophthalmol Strabismus.

[11] Xue K, Huang X, Xu S (2020). The evolution of isolated neovascular tufts (“Popcorn”) in retinopathy of prematurity. Retina.

[12] McLeod DS, D'Anna SA, Lutty GA (1998). Clinical and histopathologic features of canine oxygen-induced proliferative retinopathy. Invest Ophthalmol Vis Sci.

[13] Hartnett ME (2015). Pathophysiology and mechanisms of severe retinopathy of prematurity. Ophthalmology.

[14] de Venecia G, Jampol LM (1984). The eye in accelerated hypertension. II. Localized serous detachments of the retina in patients. Arch Ophthalmol.

[15] Pines N (1929). Sclerosis of the retinal vessels. Br J Ophthalmol.

